# Volatile Substances, Quality and Non-Targeted Metabolomics Analysis of Commercially Available Selenium-Enriched Rice

**DOI:** 10.3390/molecules29235703

**Published:** 2024-12-03

**Authors:** Yu Zhang, Qianqian Lian, Jianji Zhao, Yanping He, Huang Dai, Xiuying Liu, Wei Zhang, Jie Bi

**Affiliations:** Key Laboratory for Deep Processing of Major Grain and Oil, College of Food Science and Engineering, Wuhan Polytechnic University, Ministry of Education, Wuhan 430023, China; 15872365045@163.com (Y.Z.); 18337127754@163.com (Q.L.); 18287076058@163.com (J.Z.); heyp@whpu.edu.cn (Y.H.); huangdai9@126.com (H.D.); xiuyingliu727@126.com (X.L.); zhangwei_food@163.com (W.Z.)

**Keywords:** selenium-enriched rice, taste quality, nutritional quality, volatile components, metabolomics

## Abstract

Selenium is an essential trace element for the human body. However, its intake is usually low. Therefore, the production and utilization of selenium-enriched food are currently a research hotspot. Despite the remarkable scientific interest in this topic, only a few of the numerous studies focus on commercially available products. This study examined the nutritional quality, physical and chemical properties, cooking characteristics, and eating quality of four commercially available hot-selling rice types, both selenium-enriched and non-selenium-enriched, and discovered that selenium-enriched rice outperforms ordinary rice in terms of both nutritional quality and taste. In addition, we employed the gas chromatography–ion mobility spectrometry (GC-IMS) technique to evaluate the volatile chemicals of rice. Some of the chemicals that made selenium-rich rice taste different from regular rice were pentanal, (E)-2-Hexen-1-ol, ethyl-3-methyl butanoate, 2-furan methanol acetate, ethyl heptanoate, ethyl hexanoate, methyl hexanoate, isopentyl pentanoate, and ethyl butyrate. We looked into the metabolite profiles of rice using LC-MS-based untargeted metabolomics to obtain a better idea of the different metabolites that are found in selenium-enriched rice compared to regular rice. We identified a total of 522 metabolites and screened 182, 227, and 100 differential metabolites in selenium-enriched (A) vs. non-selenium-enriched rice (B/C/D) groups, respectively. This study revealed that selenium primarily influenced the metabolism of D-amino acids, starch, sucrose, and linoleic acid in rice. This study systematically analyzed the quality differences between selenium-enriched and non-selenium-enriched rice available on the market. For consumers, it is essential to understand the quality of selenium-rich rice on the market to guide the purchase of rice.

## 1. Introduction

Rice, scientifically known as *Oryza sativa* L., has a rich agricultural history and holds significant importance as a staple food crop worldwide. Rice serves as the primary food supply for more than 60% of the global population [1]. Consumers are placing greater emphasis on nutritional variety and quality when eating rice, resulting in an improvement in the overall standard of living. As an essential trace element, selenium participates in various biochemical reactions and plays an important role in the normal physiological activities of organisms [2]. Zhang et al. [3] have demonstrated that selenium can improve human immunity, regulate lipid metabolism, and alleviate cardiovascular diseases. A lack of selenium can result in the development of degenerative heart disease and a condition called ‘Keshan disease’ (also known as Kaschin–Beck disease), which causes deformities in the affected joints [4]. However, an overabundance of selenium can lead to hair and nail loss, gastrointestinal disorders, and potentially fatal consequences [5,6,7]. The World Health Organization (WHO) recommends that healthy adults consume 60–200 μg of selenium per day, with a maximum tolerable intake of 400 μg [8]. Therefore, the daily intake of appropriate amounts of selenium is helpful to maintain human health. Nevertheless, due to the uneven distribution of selenium in different regions, the global selenium-deficient population accounts for about 15%, while about 72% of the population in China is selenium-deficient [9]. Consuming selenium-enriched agricultural products is the most direct and effective way to supplement selenium for the majority of selenium-deficient people.

Enshi City, located in Hubei Province, China, has a wide distribution of selenium-containing soils, independent selenium deposits, and a naturally selenium-rich biosphere [10]. It is recognized as the ‘world selenium capital’. Numerous studies have demonstrated that applying foliar fertilization and irrigating rice with selenium during planting can increase the selenium content [11,12]. The physicochemical properties and cooking and eating quality of rice directly affect consumers’ nutritional quality and taste experience. However, there are relatively few comparative studies on the physicochemical properties, cooking, and eating qualities of selenium-enriched rice and ordinary rice. Furthermore, there are few reports on the differences in metabolic components between selenium-enriched rice and ordinary rice. A complex mixture of odor-active compounds composes the volatiles of rice, and storage alters their concentration [13]. Uawisetwathana and Karoonuthaisiri [14] state that metabolomics is a rapid method for identifying and quantifying tiny organic molecules (<1000 da) in live organisms. It can aid in elucidating the relationship between metabolites and phenotypic and gene expression features. Prior studies have demonstrated that metabolomics analysis technology is capable of distinguishing between rice samples originating from various regions of the world and identifying potential volatile biomarkers [15,16]. The results demonstrate that metabolomics can effectively identify the volatile distinctions between selenium-enriched rice and ordinary rice, providing comprehensive insights into metabolites.

This research aims to analyze and evaluate the physical and chemical features, cooking and eating quality, and metabolite differences between selenium-enriched rice and ordinary rice. In order to comprehensively comprehend the disparities between the two kinds of rice, we conducted an analysis of their physical and chemical properties, as well as the textures, pasting qualities, volatile compounds, and metabolomics of two distinct groups. There are few reports on the overall quality of selenium-enriched rice in the market and the comparative study of the quality of selenium-enriched rice and non-selenium-enriched rice. In this paper, the overall quality of selenium-enriched was systematically analyzed.

## 2. Results

### 2.1. Analysis of Volatile Substances

#### 2.1.1. GC-IMS Spectra of Four Kinds of Rice

**Figure 1:**
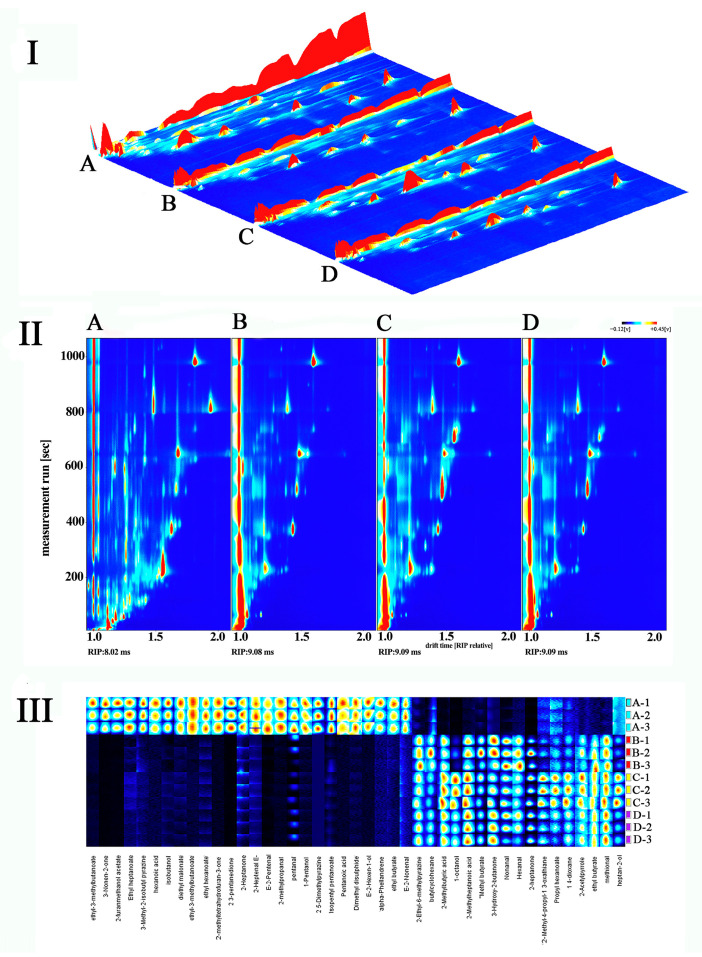
GC−IMS analysis of selenium-enriched rice and non-selenium-enriched rice; (**I**): 3D spectra; (**II**): 2D spectra; (**III**): fingerprint. A: selenium-enriched rice. B, C, D: non-selenium-enriched rice.

The aroma of rice originates from volatile chemicals, and the specific type and quantity of these molecules influence the scent of rice. The detection of volatile compounds in four groups of rice was performed using the GC-IMS technique. As shown in Figure 1(I), the three-dimensional topographic map is exported through the GC-IMS instrument plug-in. The X, Y, and Z axes represent the relative drift time, residence time, and peak intensity of ions, respectively. The spectrum displays all volatile components as distinct spots, with the color indicating the signal intensity [17,18]. In order to make a direct comparison of the variations in flavor components across different groups of rice, we employed a two-dimensional topographic map, as shown in Figure 1(II). The volatile substances of the four groups of rice were well separated by GC-IMS. We found that the volatile substances in group A were significantly different from those in groups B, C, and D, and the proportion of some flavor components in group A was higher than that in groups A, B, and C. This may be due to flavonoids, phenols, and fatty acids [19,20].

#### 2.1.2. Identification of Volatile Flavor Components in Four Kinds of Rice

A total of 43 characteristic peaks were captured from the GC-IMS two-dimensional spectra of all samples (Table S1). In order to visually analyze the variations in the specific peaks of interest across all rice samples, we created a fingerprint for comparison (Figure 1(III)). In addition, under the same conditions, we analyzed the mixed solution containing six standard compounds (2-butanone, 2-pentanone, 2-hexanone, 2-heptanone, 2-octanone, and 2-nonanone), and used the mixed solution to construct a nonlinear relationship between retention time and retention index for database connection. Then, the substances corresponding to these characteristic peaks were retrieved and identified according to the retention index and IMS database. The final qualitative identification results are shown in Table S1. As shown in Figure 1(III), the vertical axis represents the sample name, and the horizontal axis represents the volatile substance. The hue and luminosity of the signal peak correspond to the concentration of the substance, providing a comprehensive depiction of the volatile compounds in each sample and the variations in volatile compounds across samples.

A total of 43 volatile substances were detected, including 11 esters, eight aldehydes, seven heterocyclics, five alcohols, five ketones, four acids, and three others (Table S1). Alcohols and esters were the predominant volatile components found in rice fragrance. Pentanal and (E)-2-Hexen-1-ol were specifically discovered in group A (Figure 1(III)). These alcohols are mellow aromas of C4–C8 low-carbon chain alcohols, and pentanal and (E)-2-Hexen-1-ol have apple-like fruit aromas. The identified esters consist of Ethyl-3-methylbutanoate, 2-furanmethanol acetate, ethyl heptanoate, ethyl hexanoate, methyl hexanoate, isopentyl pentanoate, and ethyl butyrate. These esters are volatile compounds that contribute to the fruity scent. These flavor compounds were isolated only from group A. Groups A, B, C, and D all share several volatile chemical compounds, including butylcyclohexane, 2-methyl-4-propyl-1,3-oxathiane, propyl hexanoate, 1,4-dioxane, and heptan-2-ol. In summary, the four kinds of rice not only contain common volatile organic compounds but also have their own unique flavor components. The flavor components of rice in groups B, C, and D are similar, and the volatile components of rice in group A are significantly different from those in groups B, C, and D.

According to the results of the GC-IMS study, it was found that the volatile substances of group A selenium-enriched rice were different from the other three non-selenium-enriched rice groups B, C, and D. We next explored whether there were differences in the quality of the four rice groups.

### 2.2. Rice Quality Evaluation

#### 2.2.1. Analysis of Rice Nutritional Quality

The nutritional composition of rice, its visual appearance, cooking characteristics, and taste profile are crucial factors in assessing the overall quality of rice. As shown in Table 1, the results demonstrate notable disparities in the levels of crude fat and moisture content between selenium-enriched rice and the control rice. The level of crude fat in selenium-enriched rice was considerably higher compared to the other three groups. Dan et al. [21] demonstrated that augmenting the selenium levels in the leaves of millet plants resulted in a substantial elevation in the crude fat content of the millet grains.

Table 1 reveals that the moisture content of selenium-enriched rice was 14.27% (group A), and there were notable variations in moisture content across the four groups. According to Wang et al. [22], rice with high selenium levels has been observed to have excessive moisture content. The protein content of selenium-enriched rice was slightly higher than that of non-selenium-enriched rice (Table 1). It was found that selenium replaced sulfur along the absorption and metabolic pathways of sulfur and combined with cysteine and methionine in protein to form selenoamino acid compounds, which were further transported to rice grains and stored in protein in the form of selenoprotein, resulting in significant differences in protein content [23]. Zeng et al. [24] discovered that applying selenium fertilizer to rice leaves during the grain-filling stage had a positive impact on the accumulation of rice protein. This is consistent with our findings. The content of ash in rice can represent the amount of mineral content in rice, representing the content of inorganic salts and trace elements, which is a reference index for evaluating food nutrition [25]. The selenium-enriched rice had the highest ash level of 1.01%. Dai et al. [26] discovered that when rice plants are sprayed with selenium fertilizer during the filling stage, a portion of the selenium is absorbed and stored in the rice grains as inorganic selenium. This could explain why selenium-rich rice has a higher ash content compared to non-selenium-rich rice.

#### 2.2.2. Analysis of Rice Pasting Properties

**Figure 2:**
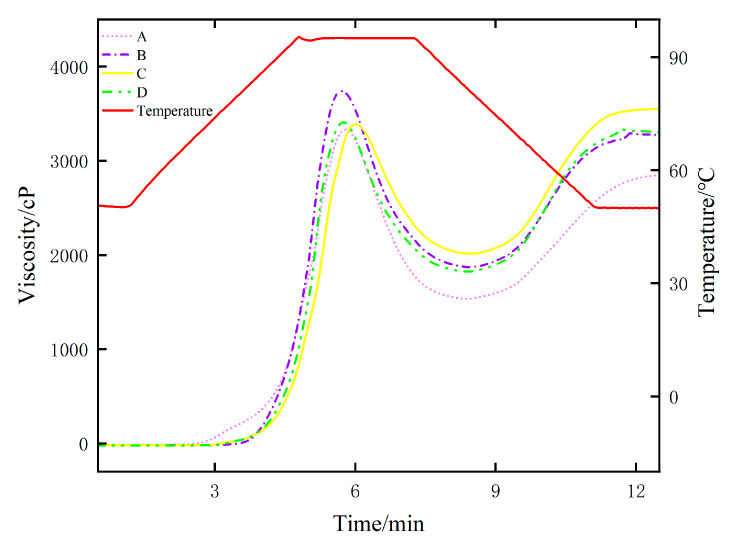
RVA profile of selenium-enriched rice and non-selenium-enriched rice. A: selenium-enriched rice. B, C, D: non-selenium-enriched rice.

The pasting curve of rice obtained by the RVA test is shown in Figure 2. The viscosity of all rice samples exhibited a consistent pattern of initially increasing, then decreasing, and finally increasing again, which is a typical RVA spectrum. The viscosity of group A and groups B, C, and D exhibited a similar trend in both pasting and retrogradation, as evident from the pasting curve.

The curve provides data on peak viscosity, holding strength viscosity, breakdown value, final viscosity, retrogradation value, and peak time. As the breakdown value increases, the thermal stability of rice paste decreases. Rice samples exhibiting high breakdown values demonstrate low shear resistance. According to Hu et al. [27], cooked rice grains are prone to breaking due to their extensive contact area with water, resulting in a favorable taste. The breakdown value observed in selenium-enriched rice is maximal (Table 2), while the breakdown values of the other samples are lower than those of group A. The holding strength viscosity can reflect the shear resistance of starch at high temperatures and affect the difficulty of food processing. The holding strength viscosity value of Se is the lowest, and the highest holding strength viscosity value of group B is 2010 cp. Consequently, selenium-enriched food is simple to process. As the retrogradation value increases, the cold paste stability of the sample decreases, the gelation becomes stronger, and the aging process becomes easier. Among all of the groups, group C exhibits the highest retrogradation value, while group A demonstrates the lowest retrogradation value.

The final viscosity can reflect the thickening ability of the sample. The final viscosity value of group C is the largest, which is significantly higher than that of group A. Chen et al. [28] demonstrated that rice with superior flavor quality tends to exhibit a higher breakdown value, lower holding strength viscosity, lower retrogradation value, and lower final viscosity when investigating its pasting qualities. Based on the aforementioned four indicators, we consider that Se rice possesses favorable eating quality.

#### 2.2.3. Analysis of Rice Texture Characteristics

The quality of rice is significantly influenced by the texture. The results of the textural characteristics of group A and groups B, C, and D under identical cooking conditions are shown in Table 3. Table 3 displays distinct variations in hardness among the different rice classes. Group A has a lower level of hardness compared to groups B and C. Groups A and B have lower amylose content compared to groups C and D (Table 1), potentially accounting for this phenomenon. Between the four types of rice, there was no discernible variation in this metric for springiness. Furthermore, group A has greater cohesiveness and springiness than groups B and D. Tasty rice is characterized by suitable hardness, springiness, and chewiness. Hard rice has poor palatability, and soft rice is highly palatable. Wang et al. [29] demonstrated that rice exhibiting lower hardness, flexibility, and chewiness displayed superior eating quality. According to the indicators provided, the flavor quality of group A outperformed the other groups in all four rice samples.

#### 2.2.4. Analysis of Rice Cooking Quality

The essence of the ripening process is the swelling and gelatinization of starch in the endosperm of rice grains. The expansion rate and water absorption rate of rice during cooking are the focus of our attention. Table 4 shows that the water absorption of all types of rice ranges from 200% to 300%, but the volume expansion ratio varies significantly. Among them, group D has the smallest volume expansion ratio, which is around 3.67 times the volume of uncooked rice. The volume expansion ratio of group C was significantly greater, nearly 4.8 times. There is a notable disparity in the rice soup dry matter content among the four groups. According to Kim et al. [30], there is a positive correlation between the amount of rice soup dry matter and the quality of the rice. There was no significant difference in the pH value of rice soup among the four groups. In addition, the iodine blue value of rice soup is used to reflect the soluble starch in rice, which has a significant correlation with the amylose content. The larger the iodine blue value, the higher the soluble amylose content in rice soup, resulting in the rice being more easily cooked. The viscosity of rice is large and relatively thick, and the taste of rice is better [12]. In Table 4, the iodine blue value in the group with Se was significantly different from groups B, C, and D, and it was like the results of rice soup dry matter.

#### 2.2.5. Analysis of Rice Taste Quality

The rice taste meter uses the near-infrared low-light transmission method to establish the relationship between the physical and chemical index content of rice and the taste. The higher the taste value, the better the rice taste [28]. The results of taste quality of the four groups of rice are shown in Table 5. The value of the appearance in group A was the highest compared to the control rice. In addition, there was a significant difference in the taste value between groups A and D, and the results of elasticity were similar. More importantly, the composite score in group A was the highest compared to groups B, C, and D, which means the eating quality of group A was the best.

#### 2.2.6. Correlation Analysis

As shown in Table 6, selenium was significantly positively correlated with the appearance, taste, composite score, and crude fat content of rice, with correlation coefficients of 0.969, 0.974, 0.956, and 0.968, respectively. It is worth noting that the correlation coefficients between rice protein and ash content and selenium content are high, reaching 0.448 and 0.426, respectively, which indicates that selenium is beneficial to the accumulation of rice protein, and a portion of selenium absorbed and utilized by rice is stored in rice grains in the form of inorganic selenium, which is consistent with the results of Liu and Ning [31]. The relationship between the selenium content and the pasting properties, textural properties, and cooking quality of rice was not statistically significant. To summarize, selenium has a beneficial impact on the nutritional composition of rice, facilitating the accumulation of rice protein and inorganic compounds.

### 2.3. Metabolomics

#### 2.3.1. Multivariate Statistical Analysis

Currently, the research on selenium-enriched and non-selenium-enriched rice primarily revolves around the analysis of well-known nutrients and bioactive components. However, there is limited reporting on the presence of unknown nutrients and active components. Metabolomics primarily involves the examination of metabolites that are generated by various metabolic pathways from substrates and products. It can thoroughly investigate alterations in metabolite composition and elucidate the variations in distinct metabolites and treatment circumstances to assess the biological responses and impacts of different substances [32,33]. We performed untargeted LC-MS-based metabolomics to investigate the metabolite profiling of group A and groups B, C, and D.

**Figure 3:**
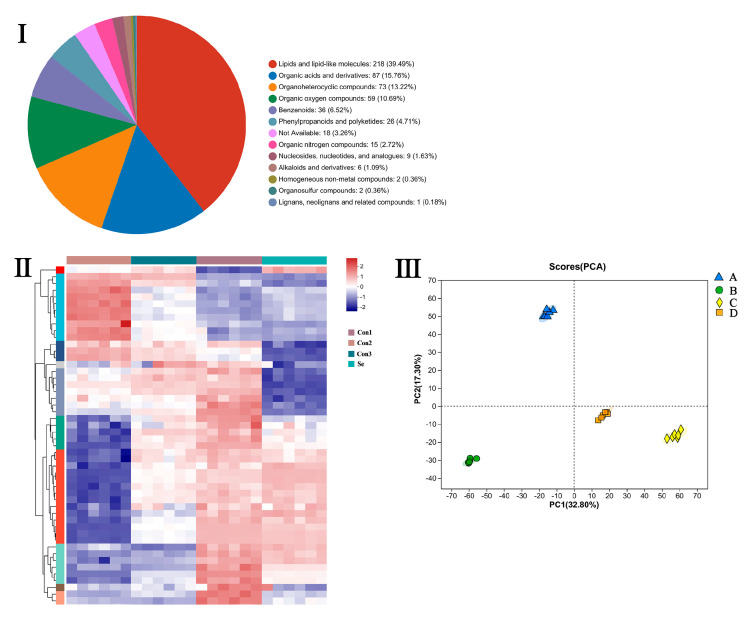
Classification, hierarchical cluster analysis (HCA), and principal component analysis (PCA) of metabolic profiles of selenium-enriched and non-selenium-enriched rice. (**I**) The classification of 522 metabolites in the sample. (**II**) Metabolite HCA map. A column represents each sample, and each metabolite is displayed in a row. Red indicates higher metabolite abundance, and blue indicates lower metabolite abundance. (**III**) PCA score map.

A total of 522 metabolites were identified, including lipids and lipid-like molecules (39.49%), organic acids and derivatives (15.76%), organoheterocyclic compounds (13.22%), organic oxygen compounds (10.69%), benzenoids (6.52%), phenylpropanoids and polyketides (4.71%), organic nitrogen compounds (2.72%), nucleosides, nucleotides, and analogs (1.63%), alkaloids and derivatives (1.09%), homogeneous non-metal compounds (0.36%), organic sulfur compounds (0.36%), lignans, neolignans, and related compounds (0.18%), and not available (3.26%) (Figure 3(I)).

Hierarchical cluster analysis (HCA) was performed, and a heat map was obtained according to the relative content difference in metabolites (Figure 3(II)). The results showed that the metabolic characteristics of the four groups of samples were significantly different. Each group had six biological replicates with good repeatability and high data reliability. The principal component analysis (PCA) score plot indicates that each group has a different metabolite profile (Figure 3(III)). The results of PCA and HCA showed that the four varieties were divided into four different groups, and there were significant differences in the metabolite profiles of each group.

#### 2.3.2. Identification of Differential Metabolites

The Orthogonal Partial Least Squares-Discriminant Analysis (OPLS-DA) model was employed to compare group A with groups B, C, and D, enabling the identification of metabolites that exhibit differential characteristics. The model had a prediction ability over 0.9, showing its appropriateness for conducting differential metabolite analysis. Group A exhibited a notable distinction from groups B, C, and D, suggesting a substantial variation in metabolic phenotype (Figure S1(IV–VI)). According to the results of the OPLS-DA model, the differential metabolites between different groups were selected: VIP ≥ 1, fold change FC ≥ 2 or FC ≤ 0.5. The results showed that there were 182 significantly different metabolites between groups A and B (Figure S1(I)), 227 between groups A and C (Figure S1(II)), and 100 between groups A and D (Figure S1(III)). The Venn diagram (Figure S1(VII)) further emphasized this observation, where the specific metabolites in each comparison showed the following order: C (227), A (182), and D (100). In three pairwise comparisons, 87 overlapping differential metabolites were key metabolites of selenium-enriched rice.

#### 2.3.3. Variable Influence on Projection (VIP) Value Analysis of Differential Metabolites

**Figure 4:**
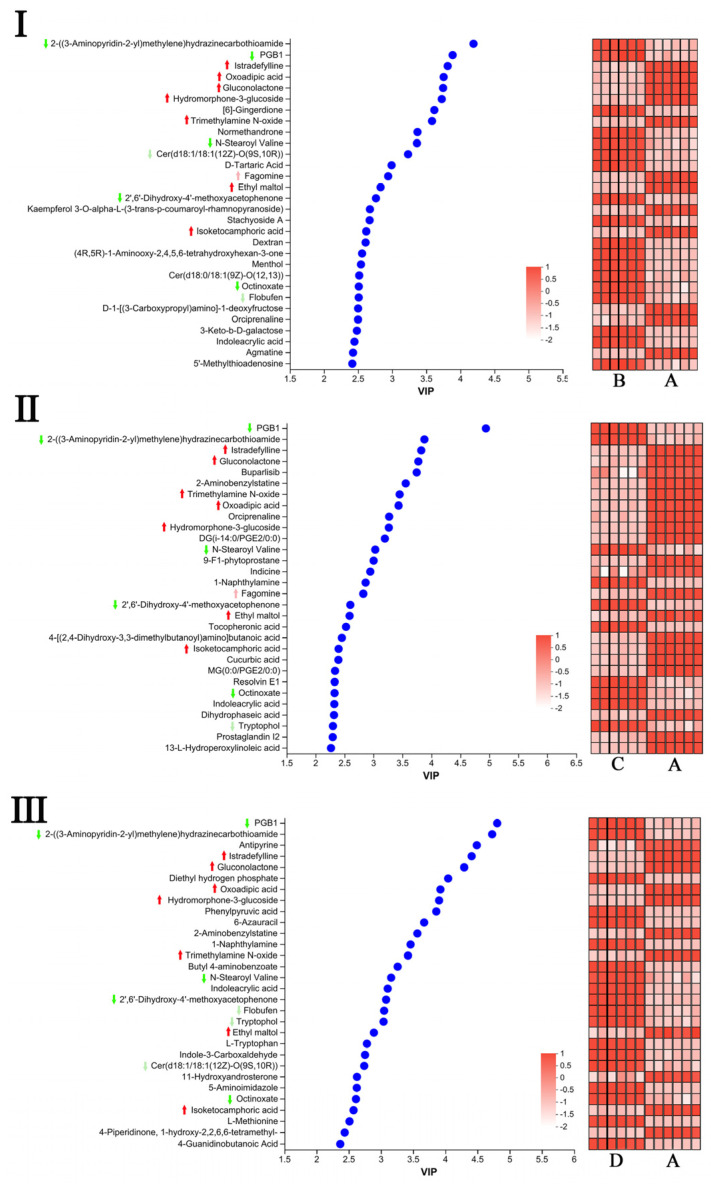
VIP value analysis of selenium-enriched rice and non-selenium-enriched rice. (**I**), A vs. B; (**II**), A vs. C; (**III**), A vs. D. Each blue dot carries a metabolite. Dark red and dark green represent the up-regulation and down-regulation of common metabolites in the three groups. Light red and light green represent the up-regulation and down-regulation of metabolites between the two groups.

To further study the effect of individual volatile compounds on the separation of different groups, the VIP score of each compound was calculated. Deep red and deep green arrowheads indicate common metabolites among the three comparison groups (Figure 4). A total of 12 species including PGB1, 2-((3-Aminopyridin-2-yl) methylene) hydrazinecarbothioamide, Istradefylline, Oxoadipic Acid, Gluconolactone, Hydromorphone-3-glucoside, N-Stearoyl Valine, Trimethylamine N-oxide, 2′,6′-Dihydroxy-4′-methoxy acetophenone, Ethyl Maltol, Octinoxate, Isoketocamphoric Acid. Among them, the deep red arrow represents seven species of substances that were higher in the group of Se, compared to the control groups, while the dark green arrow represents the opposite, with a total of five species. The light green and light red arrows in the diagram indicate that they are common differential metabolites in the two groups of comparisons.

There is one common metabolite (Fagomine) in the comparisons of A vs. B and A vs. C (Figure 4(I,II)); there are two common metabolites in the comparisons of A vs. B and A vs. D, which are Cer (d18: 1/18: 1 (12Z)-O (9S, 10R)) and Flobufen (Figure 4(I,III)); there is one common metabolite (Tryptophol) in the comparisons of A vs. C and A vs. D (Figure 4(II,III)). These findings further highlight the important differences in these metabolites between selenium-enriched rice and ordinary rice samples, which may involve specific biological processes or metabolic pathways.

#### 2.3.4. Kyoto Encyclopedia of Genes and Genomes (KEGG) Annotation and Enrichment Analysis of Differential Metabolites

**Figure 5:**
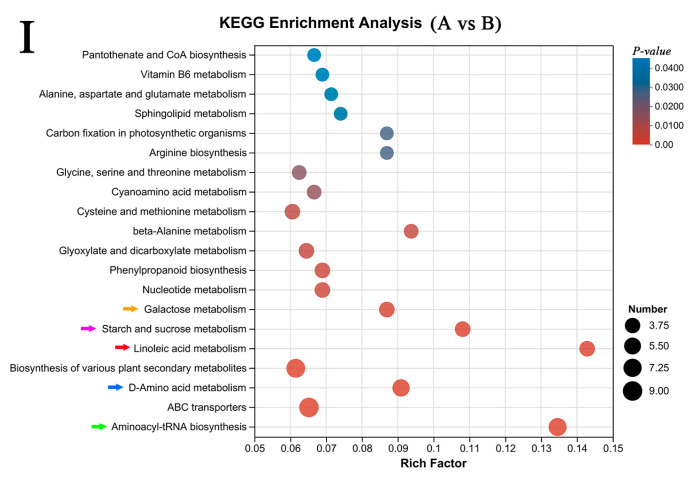

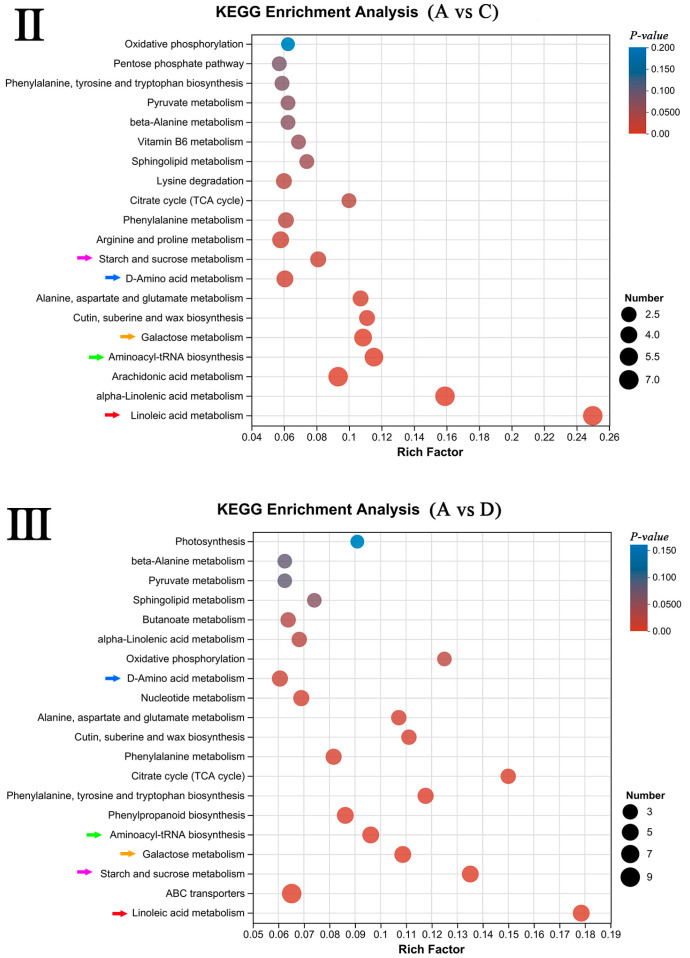
KEGG analysis of selenium-enriched rice and non-selenium-enriched rice. (**I**), A vs. B; (**II**), A vs. C; (**III**), A vs. D. Same-color arrows represent the same metabolic pathway. The larger the bubble in the figure, the more metabolites enriched on the pathway.

KEGG is used to study signal transduction pathways and metabolite accumulation. In this study, we annotated and enriched the differential metabolites of each control group and divided them into different KEGG pathways. The differential metabolites of A vs. B, C, and D, which are rich in the KEGG database, involve 57, 69, and 59 pathways, and the main pathways are represented by bubble diagrams (Figure 5). In A vs. B (Figure 5(I)), metabolic pathways such as aminoacyl-tRNA biosynthesis, ABC transporters, biosynthesis of various plant secondary metabolites, D-amino acid metabolism, and linoleic acid metabolism were significantly enriched (*p* < 0.05); In A vs. C (Figure 5(II)), significantly enriched metabolic pathways were related to linoleic acid metabolism, alpha-linolenic acid metabolism, glycerophospholipid metabolism, arachidonic acid metabolism, and glyoxylate and dicarboxylate metabolism (*p* < 0.05); in A vs. D (Figure 5(III)), metabolic pathways such as linoleic acid metabolism, aminoacyl-tRNA biosynthesis, D-amino acid metabolism, glyoxylate and dicarboxylate metabolism, and alanine, aspartate, and glutamate metabolism were significantly enriched (*p* < 0.05). The color arrows in Figure 5 are the common enrichment metabolic pathways of the three groups. We speculate that selenium may affect these five metabolic pathways, which are aminoacyl-tRNA biosynthesis, D-amino acid metabolism, linoleic acid metabolism, starch and sucrose metabolism, and galactose metabolism (*p* < 0.05). Selenium affects plants mainly through ‘amino acid biosynthesis’ and ‘starch and sucrose metabolism’. Selenium can improve the synthesis, catabolism, and utilization of amino acids and sugars, and ultimately promote plant growth [34]. Linoleic acid metabolism was also significantly enriched in the three groups. Linoleic acid is a common unsaturated fatty acid in cereals. Secondly, linoleic acid is metabolized to linoleic acid hydroperoxide (HPODE). HPODE has high activity and cytotoxicity, which can destroy important cell structures, including cell membranes and nucleic acids, and affect cell function. The hydroperoxides 13 (s) -HPODE and 9 (s) -HPODE of linoleic acid in the linoleic acid metabolic pathway showed a downward trend, indicating that Se caused the rice to accumulate more hydroperoxides to resist stress [35]. Hopode is a multifunctional intermediate that can produce aromatic compounds and bifunctional ω-oxo acids [36]. Miyazaki et al. [37] also confirmed that it is related to the taste of food.

## 3. Materials and Methods

In this study, four kinds of rice sold on the market were selected; group A is Enshi Longjian Qiyue brand rice (Enshi Selenium Food Processing Co., Ltd., Hubei, China), group B is Jinlongyu brand rice (Yihai Kerry Arawana Holdings Co., Ltd., Hubei, China), group C is Thai Tainalan brand rice (Shenzhen Guoliang Rice Industry Co., Ltd., Shenzhen, China), and group D is Cambodia Tainalan brand rice (Shenzhen Guoliang Rice Industry Co., Ltd., Shenzhen, China).

### 3.1. Main Equipment

K9840 automatic Kjeldahl nitrogen analyzer, Jinan Haineng Instrument Co., Ltd., Jinan, China; inductively Coupled Plasma Mass Spectrometer Model 7900, Agilent Inc., Santa Clara, CA, USA; TA XT plus Texture Analyzer, Stable Micro Systems, Godalming, UK; STA1B rice taste meter, Sasaki Company, Asahikawa, Japan; 7222N visible spectrophotometer, Shanghai Instrument & Electrical Analysis Instrument Co., Shanghai, China; super 4 Rapid Viscosity Analyzer, Newport Scientific Instruments, Warriewood, Australia; Flavor Spec^®^ Gas Chromatography-Ion Mobility Spectrometer G.A.S. Company, Frankfurt am Main, Germany.

### 3.2. Experimental Methods

#### 3.2.1. GC-IMS Analysis Conditions

The 5.00 g rice sample to be tested was weighed and transferred to a 20 mL headspace injection bottle. The specific detection conditions of GC-IMS were as follows: headspace conditions: the sample was heated in the form of oscillation, the incubation temperature was 90 °C, the incubation time was 10 min, the incubation speed was 500 rpm, the injection needle temperature was 90 °C, and the gas injection volume was 500 μL. GC conditions: the chromatographic column was FS-SE-54-CB-1 (15 m × 0.53 mm), the column temperature was 40 °C, the carrier gas was high-purity N_2_ (purity ≥ 99.999%), and the carrier gas flow rate was as follows: initial flow rate of 2 mL/min, maintained for 2 min, increased to 15 mL/min in 10 min, then increased to 100 mL/min in 10 min, and increased to 150 mL/min in the last 10 min, for a total running time of 30 min. IMS conditions: ion source = tritium (6.5 keV); positive ion mode; migration tube length = 9.8 cm; migration tube temperature = 45 °C; migration gas = N_2_ (purity ≥ 99.999%); migration gas flow rate = 150 mL/min; electric field intensity = 500 V/cm.

GC-IMS Library Search software 2.2.1 was used for qualitative analysis of volatile flavor compounds. The fingerprint of volatile flavor compounds was constructed by using the Reporter and Gallery Plot plug-in in Laboratory Analytical Viewer (LAV) analysis software 1.0.3.

#### 3.2.2. Physical and Chemical Properties of Rice

Detection of basic components of rice: Amylose content was determined according to GB/T15683-2008 [38], ‘*Rice-Determination of amylose content*’. The total selenium content was determined by inductively coupled plasma mass spectrometry. The determination of protein content was based on GB5009.5-2016 [39], ‘*National food safety standard-Determination of protein in food*’ (Kjeldahl method). The determination of lipid content was based on GB5009.6-2016 [40], ‘*Determination of fat in food’*. GB5009.3-2016 [41], ‘*Determination of Moisture in Food’* was also used. The determination of ash content was based on GB5009.4-2016 [42], ‘*Determination of ash content in food*’.

Cooking quality of rice: According to the experimental method of rice cooking characteristics of Shu [43], the water absorption rate, expansion volume, pH, dry matter, and iodine blue value of rice soup were determined.

Rice pasting properties (RVA) were determined according to GB/T24852-2010 [44], ‘*Determination of the pasting properties of rice-rapid visco analyzer method*’.

Texture characteristics of rice: To simulate the normal chewing state, the method of Paiva et al. [20] was used and slightly modified. Firstly, the texture analyzer was corrected, and then three grains of steamed rice were randomly selected and placed in parallel in the middle of the determination stage. TPA mode was chosen for determination. The specific determination parameters were set as follows: probe: P/36R; compression ratio: 50%; trigger force: 5 g; time interval: 5 s; pre-test speed: 1 mm/s; test speed: 2 mm/s; post-test speed: 1 mm/s.

Rice taste quality: The STA1B rice taste meter was turned on and preheated for 30 min. The black and white reference board provided was used with the instrument for calibration and testing. A total of 8.0 g of rice was weighed and gently pressed into the forming ring, the forming ring was pressed into the shaper, the handle of the shaper was pressed to the limit position and held for 10 s, the forming ring was reversed, pressing continued for 10 s, and the rice shaping was completed. The formed rice samples were placed in the measuring tank of the rice taste meter for testing. Each rice sample was measured twice in positive and negative directions. The above operation was repeated, and rice samples of each rice variety were taken 3 times. Finally, the appearance, taste, and other taste data of the sample rice were exported to the computer connected to the rice taste meter.

#### 3.2.3. Non-Targeted Metabolomics Analysis

A total of 5.00 g whole rice was sealed in a 10 mL centrifuge tube and sampled six times in parallel. The samples were frozen in liquid nitrogen and stored at 80 °C for testing. The samples were submitted to Shanghai Meiji Biomedical Technology Co., Ltd. (Shanghai, China) for non-targeted metabolomics detection.

Rice samples of 50 mg were centrifuged in 2 mL centrifuge tubes, and a grinding ball with a diameter of 6 mm was added. The 400 μL extract (methanol/water = 4:1 (*v*:*v*)) containing 0.02 mg/mL internal standard (L-2-chlorophenylalanine) was used for metabolite extraction. The sample solution was ground in a frozen tissue grinder for 6 min (−10 °C, 50 Hz), followed by low-temperature ultrasonic extraction for 30 min (5 °C, 40 kHz). The sample was placed at 20 °C for 30 min and centrifuged for 15 min (4 °C, 13,000× *g*), and the supernatant was transferred to a vial with an intubating tube for analysis. After obtaining the data, we used the Meiji online cloud platform for subsequent analysis and processing (https://www.majorbio.com/).

#### 3.2.4. Data Processing and Analysis

Statistical analysis and collation of the data were performed using Excel 2021 statistical software. IBM SPSS Statistics 26.0 data analysis software was used to perform variance analysis and correlation analysis on the data. The LSD test was selected for variance analysis. Statistical analysis was performed on the data at the *p* < 0.05 test level. The results were expressed as mean ± standard deviation. The drawing was completed by Origin 2022.

## 4. Conclusions

This study aimed to compare the nutritional quality, physicochemical qualities, cooking characteristics, and eating quality of one kind of selenium-enriched rice and three types of non-selenium-enriched rice. Furthermore, we analyzed the volatile components and their metabolic processes. The findings indicated that the enrichment of selenium in rice promoted an increase in protein and ash content, and enhanced the overall quality and taste of rice. However, there were no notable variations in the texture or cooking characteristics of the rice.

The GC-IMS technique was used to analyze the volatile chemicals present in both selenium-enriched and ordinary rice. A total of 43 volatile compounds were identified, consisting of 11 esters, eight aldehydes, seven heterocycles, five alcohols, five ketones, four acids, and three miscellaneous chemicals. In addition, we employed untargeted LC-MS-based metabolomics to examine the metabolite profiles of Se-enriched and ordinary rice. A total of 522 compounds with significant changes were detected, including lipids, organic acids, organic heterocycles, aerobic compounds, benzenes, phenylacetones, and polyketides. By conducting KEGG annotation and enrichment analysis on differential metabolites, it was determined that selenium primarily impacts three metabolic pathways: D-amino acid, starch and sucrose, and linoleic acid metabolism. This study provides new ideas for the product development of selenium-enriched rice and for improving the flavor quality of rice. It also provides a reference for consumers to go to the market to buy selenium-enriched and non-selenium-enriched rice.

## Figures and Tables

**Table 1:** Nutritional quality analysis of selenium-enriched and non-selenium-enriched rice.

Group	Amylose (wt%)	Protein (wt%)	Crude Fat (wt%)	Moisture Content (wt%)	Ash Content (wt%)	Selenium Content (mg/kg)
A	12.57 ± 0.42 ^a^	9.03 ± 0.12 ^a^	0.94 ± 0.05 ^a^	14.27 ± 0.06 ^d^	1.01 ± 0.03 ^a^	0.25 ± 0.01 ^a^
B	13.59 ± 0.34 ^ab^	8.83 ± 0.07 ^a^	0.86 ± 0.01 ^b^	13.70 ± 0.10 ^c^	0.98 ± 0.01 ^a^	0.09 ± 0.00 ^b^
C	13.73 ± 0.21 ^ab^	8.97 ± 0.06 ^a^	0.84 ± 0.03 ^b^	12.70 ± 0.00 ^a^	0.90 ± 0.01 ^b^	0.08 ± 0.01 ^b^
D	14.37 ± 0.72 ^b^	8.01 ± 0.65 ^b^	0.83 ± 0.01 ^b^	13.53 ± 0.06 ^b^	0.87 ± 0.02 ^b^	0.09 ± 0.00 ^b^

Note: Results are the average of duplicate measurements ± SD. Mean values in the same column with different letters are significantly different (*p* < 0.05).

**Table 2:** Analysis of pasting properties of selenium-enriched and non-selenium-enriched rice.

Group	Peak Viscosity/cP	Holding Strength Viscosity/cP	Retrogradation Value/cP	Final Viscosity/cP	Breakdown Value/cP	Peak Time/min
A	3483.67 ± 195.81 ^a^	1668.67 ± 184.37 ^b^	1355.33 ± 37.65 ^b^	3024.00 ± 207.94 ^c^	1815.00 ± 14.93 ^a^	5.89 ± 0.10 ^ab^
B	3616.67 ± 137.76 ^a^	1850.00 ± 19.05 ^ab^	1382.00 ± 24.76 ^b^	3232.00 ± 40.04 ^b^	1766.67 ± 123.57 ^a^	5.76 ± 0.10 ^bc^
C	3423.67 ± 39.43 ^a^	2010.00 ± 15.39 ^a^	1527.33 ± 29.48 ^a^	3537.33 ± 44.84 ^a^	1413.67 ± 52.35 ^b^	6.00 ± 0.00 ^a^
D	3508.67 ± 156.46 ^a^	1801.67 ± 29.87 ^b^	1491.00 ± 20.42 ^a^	3292.67 ± 37.54 ^b^	1707.00 ± 152.05 ^a^	5.69 ± 0.03 ^c^

Note: Results are the average of duplicate measurements ± SD. Mean values in the same column with different letters are significantly different (*p* < 0.05).

**Table 3:** Analysis of texture characteristics of selenium-enriched and non-selenium-enriched rice.

Group	Hardness (N)	Chewiness (N)	Cohesiveness (Ratio)	Resilience (Ratio)	Springiness (Ratio)
A	6.67 ± 0.93 ^b^	1.96 ± 0.52 ^ab^	0.50 ± 0.04 ^a^	0.35 ± 0.03 ^a^	0.61 ± 0.05 ^a^
B	6.81 ± 0.33 ^b^	1.60 ± 0.23 ^b^	0.45 ± 0.03 ^b^	0.35 ± 0.03 ^a^	0.54 ± 0.03 ^b^
C	8.81 ± 0.99 ^a^	2.46 ± 0.55 ^a^	0.47 ± 0.04 ^ab^	0.35 ± 0.03 ^a^	0.59 ± 0.04 ^ab^
D	8.28 ± 1.13 ^a^	2.00 ± 0.57 ^ab^	0.44 ± 0.05 ^b^	0.34 ± 0.05 ^a^	0.54 ± 0.05 ^b^

Note: Results are the average of duplicate measurements ± SD. Mean values in the same column with different letters are significantly different (*p* < 0.05).

**Table 4:** Cooking characteristics of selenium-enriched and non-selenium-enriched rice.

Group	Water Absorption (%)	Volume Expansion Ratio (%)	Rice Soup Dry Matter (%)	Rice Soup pH	Iodine Blue Value
A	254.56 ± 1.95 ^a^	468.23 ± 2.36 ^b^	85.80 ± 1.71 ^a^	7.51 ± 0.36 ^a^	0.417 ± 0.01 ^a^
B	242.97 ± 2.30 ^ab^	389.23 ± 3.56 ^c^	55.53 ± 2.00 ^b^	7.24 ± 0.11 ^a^	0.236 ± 0.03 ^c^
C	240.84 ± 3.48 ^ab^	479.32 ± 1.23 ^a^	57.73 ± 1.86 ^b^	7.62 ± 0.25 ^a^	0.322 ± 0.02 ^b^
D	240.60 ± 3.40 ^b^	367.15 ± 1.09 ^d^	41.87 ± 3.51 ^c^	7.24 ± 0.45 ^a^	0.248 ± 0.01 ^c^

Note: Results are the average of duplicate measurements ± SD. Mean values in the same column with different letters are significantly different (*p* < 0.05).

**Table 5:** Analysis of eating quality of selenium-enriched and non-selenium-enriched rice.

Group	Appearance	Tasted	Rice Elasticity	Rice Taste Value
A	7.55 ± 0.21 ^a^	6.60 ± 0.14 ^a^	0.90 ± 0.04 ^a^	77.50 ± 2.12 ^a^
B	5.65 ± 0.21 ^b^	5.45 ± 0.35 ^ab^	0.83 ± 0.08 ^a^	62.50 ± 0.71 ^b^
C	5.75 ± 0.35 ^b^	5.60 ± 0.28 ^ab^	0.88 ± 0.03 ^a^	65.50 ± 2.12 ^b^
D	6.05 ± 0.35 ^b^	5.20 ± 0.71 ^b^	0.67 ± 0.06 ^b^	59.00 ± 4.24 ^b^

Note: Results are the average of duplicate measurements ± SD. Mean values in the same column with different letters are significantly different (*p* < 0.05).

**Table 6:** Correlation of selenium with physicochemical properties and cooking flavor quality of rice.

Nutritional Quality		Pasting Properties		Texture Properties		Cooking Characteristics		Taste Quality	
Index	Correlation Coefficient	Index	Correlation Coefficient	Index	Correlation Coefficient	Index	Correlation Coefficient	Index	Correlation Coefficient
Amylose	0.123	Peak Viscosity	−0.155	Hardness	−0.647	Water Absorption	0.828	Appearance	0.969 *
Protein	0.448	Holding Strength Viscosity	−0.809	Chewiness	−0.140	Volume Expansion Ratio	0.521	Tasted	0.974 *
Crude Fat	0.968 *	Breakdown Value	0.564	Cohesiveness	0.849	Rice Soup Dry Matter	0.943	Rice Elasticity	0.427
Moisture Content	0.777	Final Viscosity	−0.815	Resilience	0.298	Rice Soup pH	0.383	Composite Score	0.956 *
Ash Content	0.426	Retrogradation Value	−0.698	Springiness	0.728	Iodine Blue Value	0.891		
		Peak Time	0.214						

Note: * indicates significant correlation (*p* < 0.05).

## Data Availability

The original contributions presented in this study are included in the article/Supplementary Materials. Further inquiries can be directed to the corresponding author.

## Supplementary Materials

The following supporting information can be downloaded at: https://www.mdpi.com/article/10.3390/molecules29235703/s1, Figure S1: Metabolite difference analysis and Venn diagram of selenium-enriched and non-selenium-enriched rice; Table S1: List of compounds characterised by GC-IMS for selenium-enriched and non-selenium-enriched rice.
