# Supplementary material for: Volatile Substances, Quality and Non-Targeted Metabolomics Analysis of Commercially Available Selenium-Enriched Rice
Source: Molecules. 2024 Dec 3;29(23):5703. doi: 10.3390/molecules29235703 (PMC11643911; doi:10.3390/molecules29235703)
Supplement: Supplementary file 1 [file molecules-29-05703-s001.zip › molecules-3325816-supplementary.pdf]

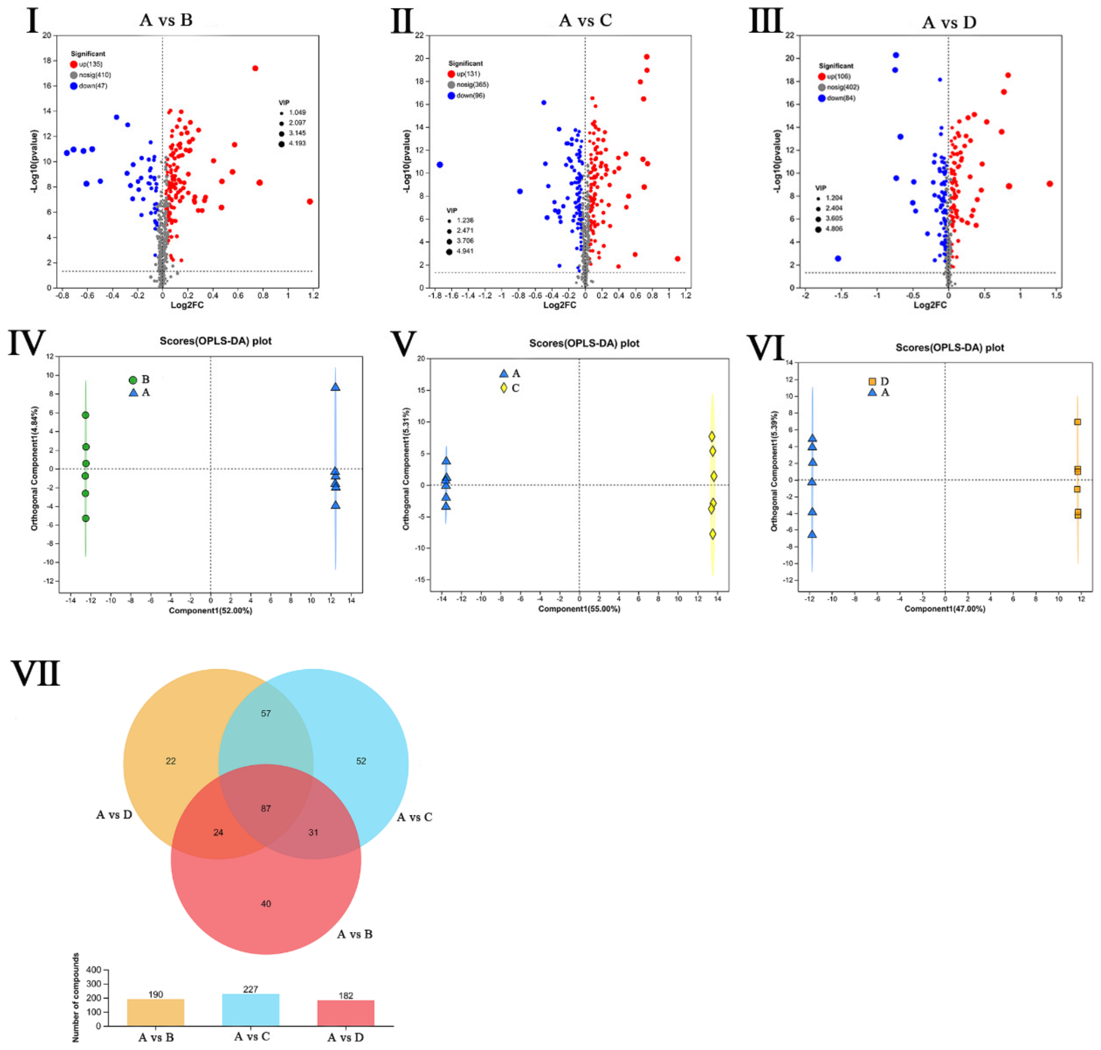

Figure S1. Metabolite difference analysis and Venn diagram of selenium-enriched and non-selenium-enriched rice.A vs B (Ⅰ), A vs C (Ⅱ) and A vs D (Ⅲ) generated by OPLS-DA. The volcano plot showed differences in the expression levels of metabolites between A vs B (Ⅳ), A vs C (Ⅴ), and A vs D (Ⅵ). (Ⅶ) Venn diagram illustrates the overlap and specific differential metabolites of the three comparison groups (A vs B; A vs C, A vs D).

Table S1. List of compounds characterised by GC-IMS for selenium-enriched and non-selenium-enriched rice.

No	Compound	CAS	Formula	Retention index	Retention time [sec]	Drift time[RIP relative]
1	ethyl-3-methylbutanoate	C108645	$C_7H_{14}O_2$	1053	523.924	1.6644
2	3-Nonen-2-one	C14309570	$C_9H_{16}O$	1134.8	815.333	1.9407
3	2-furan methanol acetate	C623176	$C_7H_8O_3$	991.3	375.252	1.628
4	Ethyl heptanoate	C106309	$C_9H_{18}O_2$	1086.1	626.696	1.4142
5	3-Methyl-2-isobutyl pyrazine	C13925069	$C_9H_{14}N_2$	1117.8	743.742	1.331
6	hexanoic acid	C142621	$C_6H_{12}O_2$	998.1	389.32	1.6574
7	isobutanol	C78831	$C_4H_{10}O$	1078.1	600.151	1.1723
8	diethyl malonate	C105533	$C_7H_{12}O_4$	1076.1	593.61	1.2498
9	ethyl-3-methylbutanoate	C108645	$C_7H_{14}O_2$	1053.6	525.532	1.254
10	ethyl hexanoate	C123660	$C_8H_{16}O_2$	1003.6	401.041	1.3392
11	2-methyltetrahydrofuran-3-one	C3188009	$C_5H_8O_2$	798.5	117.608	1.4191
12	2,3-pentanedione	C600146	$C_5H_8O_2$	692.8	55.872	1.243
13	2-Heptanone	C110430	$C_7H_{14}O$	872	194.707	1.2579
14	(E)-2-Heptenal	C18829555	$C_7H_{12}O$	940.3	287.253	1.261
15	(E)-2-Pentenal	C1576870	C_5H_8O	741	78.524	1.1128
16	2-methyl propanal	C78842	C_4H_8O	801.5	120.04	1.291
17	pentanal	C110623	$C_5H_{10}O$	692.5	55.769	1.178
18	1-Pentanol	C71410	$C_5H_{12}O$	779.5	103.032	1.244
19	2,5-Dimethylpyrazine	C123320	$C_6H_8N_2$	907.6	241.984	1.5602
20	Isopentyl pentanoate	C2050091	$C_{10}H_{20}O_2$	1139.1	834.518	1.481
21	Pentanoic acid	C109524	$C_5H_{10}O_2$	890.6	221.149	1.526
22	Dimethyl disulphide	C624920	$C_2H_6S_2$	1079	603.083	1.1541
23	(E)-2-Hexen-1-ol	C928950	$C_6H_{12}O$	875.5	199.395	1.513
24	alpha-Phellandrene	C99832	$C_{10}H_{16}$	1000.3	394.005	1.694
25	ethyl butyrate	C105544	$C_6H_{12}O_2$	1051.9	520.825	1.563
26	(E)-2-Nonenal	C18829566	$C_9H_{16}O$	1162.3	946.053	1.4119
27	2-Ethyl-6-methyl pyrazine	C13925036	$C_7H_{10}N_2$	993.6	379.999	1.203
28	butyl cyclohexane	C1678939	$C_{10}H_{20}$	1077.8	599.073	1.0359
29	2-Methylbutyric acid	C116530	$C_5H_{10}O_2$	903.8	237.2	1.211
30	1-octanol	C111875	$C_8H_{18}O$	1054.2	527.332	1.4708
31	2-Methylheptanoic acid	C1188029	$C_8H_{16}O_2$	1135.7	819.375	1.3939
32	Methyl butyrate	C623427	$C_5H_{10}O_2$	991.3	375.278	1.4379
33	3-Hydroxy-2-butanone	C513860	$C_4H_8O_2$	714.7	65.23	1.0687
34	Nonanal	C124196	$C_9H_{18}O$	1094.1	654.147	1.4876
35	Hexanal	C66251	$C_6H_{12}O$	1093.3	651.339	1.558
36	2-heptanone	C110430	$C_7H_{14}O$	1169.7	984.821	1.6038
37	2-Methyl-4-propyl-1,3-oxathiane	C67715804	$C_8H_{16}OS$	1134.8	815.527	1.7209
38	Propyl hexanoate	C626777	$C_9H_{18}O_2$	1092.8	649.754	1.8565
39	1,4-dioxane	C123911	$C_4H_8O_2$	1086.5	627.935	1.3347
40	2-Acetylpyrrole	C1072839	C_6H_7NO	1076.6	595.216	1.1023

41	ethyl butyrate	C105544	$C_6H_{12}O_2$	1061.1	547.286	1.2058
42	methional	C3268493	C_4H_8OS	901.7	234.547	1.094
43	heptan-2-ol	C543497	$C_7H_{16}O$	898.7	230.861	1.3857
